# Is meat consumption associated with depression? A meta-analysis of observational studies

**DOI:** 10.1186/s12888-017-1540-7

**Published:** 2017-12-28

**Authors:** Yi Zhang, Ye Yang, Ming-sheng Xie, Xiang Ding, Hui Li, Zhi-chen Liu, Shi-fang Peng

**Affiliations:** 1 Department of Infectious Disease, Xiangya Hospital, Central South University, Changsha, Hunan Province 410008 China; 2 Department of Orthopaedics, Xiangya Hospital, Central South University, Changsha, Hunan Province 410008 China; 3 Hunan Key Laboratory of Joint Degeneration and Injury, Central South University, Changsha, Hunan Province 410008 China; 4 Hunan Clinical Research Center of Joint Surgery, Central South University, Changsha, Hunan Province 410008 China

**Keywords:** Meat, Depression, Meta-analysis, Observational studies

## Abstract

**Background:**

A number of epidemiological studies have examined the effect of meat consumption on depression. However, no conclusion has been reached. The aim of this study was to examine the relationship between meat consumption and depression.

**Methods:**

The electronic databases of PUBMED and EMBASE were searched up to March 2017, for observational studies that examined the relationship between meat consumption and depression. The pooled odds ratio (OR) for the prevalence of depression and the relative risk (RR) for the incidence of depression, as well as their corresponding 95% confidence interval (CI), were calculated respectively (the highest versus the lowest category of meat consumption).

**Results:**

A total of eight observational studies (three cross-sectional, three cohort and two case-control studies) were included in this meta-analysis. Specifically, six studies were related to the prevalence of depression, and the overall multi-variable adjusted OR suggested no significant association between meat consumption and the prevalence of depression (OR = 0.89, 95% CI: 0.65 to 1.22; *P* = 0.469). In contrast, for the three studies related to the incidence of depression, the overall multi-variable adjusted RR evidenced an association between meat consumption and a moderately higher incidence of depression (RR = 1.13, 95% CI: 1.03 to 1.24; *P* = 0.013).

**Conclusions:**

Meat consumption may be associated with a moderately higher risk of depression. However, it still warrants further studies to confirm such findings due to the limited number of prospective studies.

**Electronic supplementary material:**

The online version of this article (10.1186/s12888-017-1540-7) contains supplementary material, which is available to authorized users.

## Background

Depression is a common mental disorder in general population, which usually presents with symptoms of sadness, exhaustion and lack of interest in daily life activities [1]. Depression may cause great losses to the society, such as the reduction in work productivity and quality of life, as well as the increase in suicide rate [2]. According to the data released by WHO, over 350 million people suffer from depression worldwide and the prevalence is rising globally [3]. The current treatments for depression may bring about a series of issues such as costly pharmacotherapy, adverse side effects and unsatisfactory curative effect [4, 5]. Therefore, alternative treatment and prevention strategy for depression are needed. There is an increasing collection of evidence to suggest that dietary factors are associated with depression [6]. Thus, the identification of modifiable dietary factors for depression appears to be an important step in the prevention and management of depression.

As a major source of protein, fat and energy for human, meat consumption accounts for a large part of the dietary structure worldwide [7]. In addition, meat contains an abundant variety of essential nutrients such as iron, zinc, and vitamin B12 [8]. Nevertheless, the 2005 US Dietary Guidelines recommend that the consumption of meat should be moderated appropriately [9], since some epidemiological evidences showed that meat consumption was associated with digestive system disease [10], cardiovascular disease [11], type 2 diabetes [12] and cancer [13]. Meat consumption is directly associated with obesity [14], which is a risk factor for depression [15]. In another word, it seems naturally to speculate that meat consumption is probably associated with depression. To our best knowledge, a number of epidemiological studies have examined the effect of meat consumption on depression [16–23]. Some of them confirmed the association between meat consumption and depression [17–19, 22], while some others rejected it [16, 20, 21, 23]. Thus, the present meta-analysis of observational studies aimed at investigating the relationship between meat consumption and depression. It was hypothesized that meat consumption is positively associated with depression.

## Methods

### Search strategy

This current meta-analysis was conducted according to the Preferred Reporting Items for Systematic review and Meta-analyses (PRISMA) guidelines [24]. The electronic databases of PUBMED and EMBASE were searched up to March 2017. The search terms used for the study selection were ‘meat’, ‘fresh’ or ‘dietary pattern’ combined with ‘depression’, ‘depressive symptom’, ‘depressive disorder’, ‘major depressive disorder’, ‘dysthymia’ or ‘mood disorder’. No language restrictions were set in the search strategy. Moreover, we reviewed the reference lists from retrieved articles to identify additional studies.

### Study selection

The titles, abstracts and full texts of all retrieved studies were reviewed independently by two researchers (YZ and SFP). Disagreements were resolved by discussions and mutual-consultations. The included studies were required to meet the following criteria: 1) observational studies (case-control, cohort or cross-sectional study); 2) the exposure of interest was meat consumption; 3) The outcome of interest were the odds ratio (OR) for the prevalence of depression and the relative risk (RR) for the risk of depression. The exclusion criteria were as follows: 1) duplicated or irrelevant articles; 2) reviews, letters, case reports; 3) non-human studies.

### Data extraction

The following information was collected independently by two researchers (YZ and SFP): first author, year of publication, location, age, gender, sample size, study design, adjustments and assessment of depression. The outcome of interest were the OR for the prevalence of depression and the RR for the risk of depression, for the highest versus the lowest category of meat consumption. The most multivariable adjusted OR and RR values reported in the original study were extracted. It is noteworthy that a cohort study also reported OR for depression at baseline and both OR and RR were extracted [20].

### Quality assessment

Quality assessment was conducted according to the Newcastle–Ottawa (NOS) criteria for non-randomized studies [25], which is based on three broad perspectives: the selection process of study cohorts, the comparability among different cohorts, and the identification of either the exposure or outcome of study cohorts [26]. Disagreements with respect to the methodological quality were resolved by discussion and mutual-consultation.

### Statistical analyses

The OR and RR for depression were the outcome measures investigated in this meta-analysis. The pooled OR, RR for depression and their related 95% confidence interval (CI) were calculated. The homogeneity of effect size across trials was tested by Q statistics (*p* < 0.05 was considered heterogeneous). If significant heterogeneity was observed among studies, the random-effects model was used; otherwise, the fixed effects model was acceptable. The I^2^ statistic, which measures the percentage of the total variation across studies due to heterogeneity, was also examined (I^2^ > 50% was considered heterogeneity). The publication bias was estimated by Begg’s tests [27], and all the statistical analyses were conducted with STATA version 11.0 (StataCorp LP, College Station, Texas). A *p* value ≤0.05 was accepted as statistically significant, unless otherwise specified [26].

## Results

### Literature search and study characteristics

**Fig. 1:**
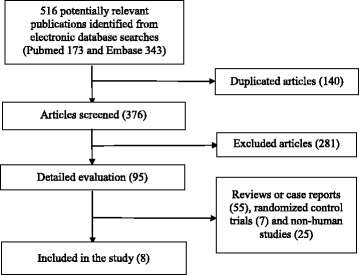
Flow chart for the identification of studies that were included in this meta-analysis

The process of identification and study selection was summarized in Fig. 1. A total of 5 hundred and sixteen potentially relevant articles (1 hundred and seventy-three in PUBMED, three hundred and forty-three in EMBASE) were identified from the initial literature search. After removing 1 hundred and forty duplicated articles, 3 hundred and seventy-six articles were evaluated in detail. Then, 2 hundred and eight-one studies were excluded based on the review of titles and abstracts initially. Fifty-five reviews and case reports, seven randomized control trials, twenty-five non-human studies were excluded. Eventually, eight studies were qualified for meta-analysis. The main characteristics of these eight studies were summarized in Table 1. Their methodological quality was shown in Additional file 1: Table S1 (cross-sectional study), Additional file 1: Table S2 (cohort study) and Additional file 1: Table S3 (case-control study) respectively.

**Table 1:** Characteristics of the individual studies included in this systematic review and meta-analysis

First author year of publication	Location	Age years	Male (%)	Sample Size	Study design	Exposure definition	OR or RR for depression (95%CI)	Adjustments	Assessment of Depression
Chen 2005 [16]	China	≥60	47.1	1600	Cross-sectional	Never	1.00	Gender, current family income, watching television, relationships with neighbors, living with whom, self-assessed physical, health status, hypertension, adverse life events occurring in the past 2 years	Geriatric Mental State and the Automated Geriatric Examination for Computer Assisted Taxonomy
						< 1 servings/week	1.76 (0.96–3.22)		
						≥1 servings/week	0.80 (0.30–2.11)		
Sanchez-Villegas 2009 [17]	Spain	37.2	41.6	10,094	Cohort	Quintile 1	1.00	Gender, age, smoking status, body mass index, physical activity during leisure time, energy intake and employment status.	A self-reported physician-made diagnosis of depression
						Quintile 2	0.92 (0.67–1.26)		
						Quintile 3	0.98 (0.72–1.32)		
						Quintile 4	1.14 (0.84–1.53)		
						Quintile 5	1.35 (1.01–1.80)		
Tsai 2011 [18]	Taiwan	≥65	57.6	1609	Cohort	< 3 servings/week	1.00	Age, gender, years of formal education, satisfaction with economic status, living setting, smoking status, alcohol drinking, betel-nut chewing, functional status, physical activity, cognitive status (SPMSQ score) and the presence of major chronic co-morbidities (hypertension, diabetes, heart disease, cancer, stroke, chronic kidney disease, gout, joint pain/arthritis, gallbladder/liver disease, hip fracture and lower-back pain)	Center for Epidemiologic Studies Depression Rating Scale (Score ≥ 10)
						≥ 3 servings/week	1.31 (0.90–1.91)		
Park 2012 [19]	Korea	44.85 ± 1.77	33.3	166	Case-control	≤0.93 servings/week	1.00	Drinking, marital status, sleeping hours, education, job and energy except for energy intake	Center for Epidemiologic Studies Depression Scale (Scores ≥25)
		43.47 ± 1.43							
						0.93–2.44 servings/week	1.18 (0.43–3.20)		
						2.44–3.61 servings/week	1.17 (0.38–3.66)		
						>3.61 servings/week	4.39 (1.25–15.38)		
Miyake 2013 [21]	Japan	31.2 ± 4.3	0	1745	Cross-sectional	Quintile 1	1.00	Age, gestation, region of residence, number of children, family structure, history of depression, family history of depression, smoking, secondhand smoke exposure at home and at work, job type, household income, education and body mass index.	Center for Epidemiologic Studies Depression Scale (Scores ≥16)
						Quintile 2	0.67 (0.47–0.96)		
						Quintile 3	1.06 (0.75–1.49)		
						Quintile 4	0.90 (0.64–1.28)		
Rienks 2013 [20]	Australia	50–55	0	8369	Cohort	Never, ever	OR	Energy, smoking, physical activity, ability to manage on available income, occupation status, education level, marital status, mean stress score and body mass index.	Center for Epidemiologic Studies Depression Scale (Scores ≥10)
							1.00		
							1.06 (0.99–1.13)		
						Never, ever	RR		
							1.00		
							1.09 (0.98–1.21)		
Zhou 2014 [22]	China	≥65	46.4	11,473	Cross-sectional	Rarely	1.00	Not mentioned	Patient Health Questionnaire-9; (Scores ≥10)
						< 250 g/week	0.61 (0.47–0.78)		
						250–500 g/week	0.41 (0.32–0.52)		
						≥ 500 g/week	0.61 (0.47–0.78)		
Kim 2015 [23]	Korea	12–18	0	849	Case-control	≤2.6 servings/week	1.00	Energy intake	Beck Depression Inventory (Scores ≥16)
						2.6–6.8 servings/week	0.82 (0.50–1.34)		
						>6.8 servings/week	0.70 (0.41–1.21)		

### Association of meat consumption and the prevalence of depression

**Fig. 2:**
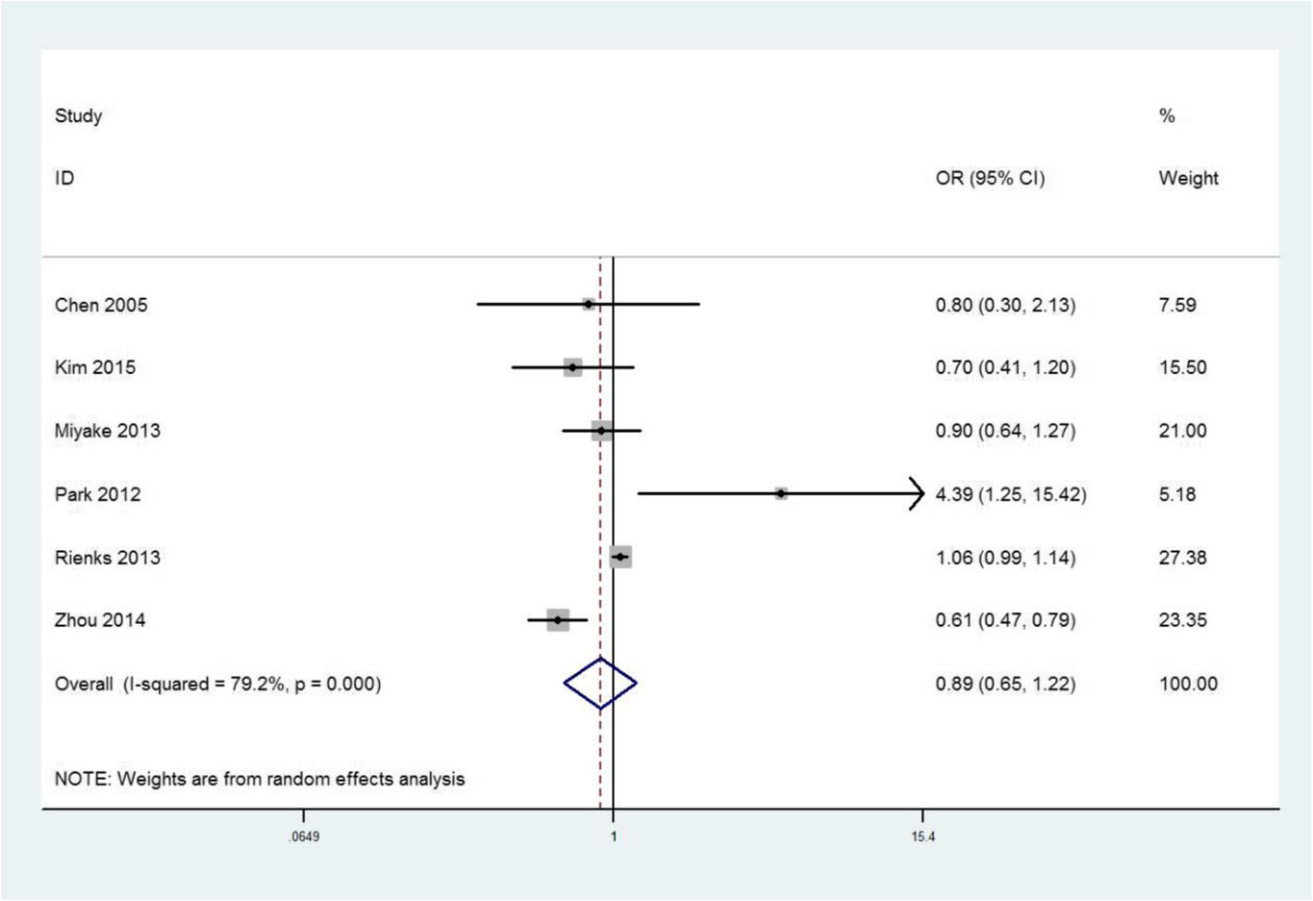
Forest plot of meta-analysis: Overall multi-variable adjusted OR of depression for the highest versus the lowest category of meat consumption

Six studies including three cross-sectional [16, 21, 22], one cohort [20] and two case-control [19, 23] studies, reported the OR of the prevalence of depression for the highest versus the lowest meat consumption category. These studies were originated from China, Korea, Japan and Australia. In terms of the study setting, two hospital-based and four community-based studies were included. The overall multi-variable adjusted OR showed that there was no relationship between meat consumption and the prevalence of depression (OR = 0.89, 95%CI: 0.65 to 1.22; *P* = 0.469) (Fig. 2). However, a substantial level of heterogeneity was found among various studies (*P* < 0.001, I^2^ = 79%). The Begg rank-correlation test showed no evidence of publication bias (*P* = 0.707).

### Association of meat consumption and the risk of depression

**Fig. 3:**
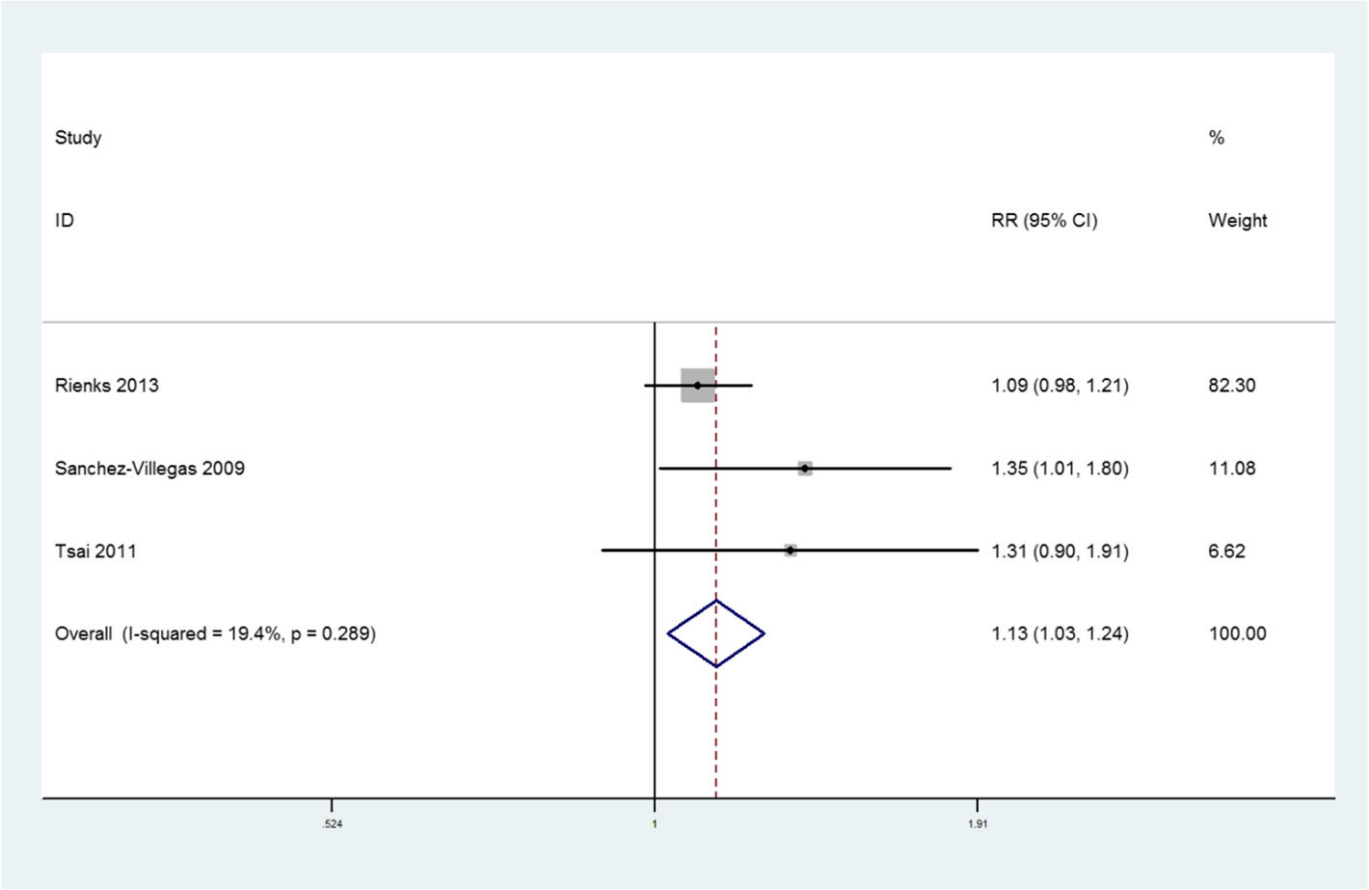
Forest plot of meta-analysis: Overall multi-variable adjusted RR of depression for the highest versus the lowest category of meat consumption

Three prospective cohort studies [17, 18, 20] reported the RR of depression for the highest versus the lowest meat consumption category. They were originated from Spain, Australia and Taiwan, which were all community-based studies. The overall multi-variable adjusted RR showed that meat consumption was associated with a moderately higher risk of depression (RR = 1.13, 95%CI: 1.03 to 1.24; *P* = 0.013) (Fig. 3). No significant heterogeneity was found among various studies (*P* = 0.289, I^2^ = 19%). The Begg rank-correlation test showed no evidence of publication bias (*P* = 1.000).

## Discussions

In the current meta-analysis, a total of eight observational studies were identified for examination. The quantitative synthesis of these observational studies showed that meat consumption might be associated with a moderately higher risk of depression. However, with respect to the prevalence of depression, no significant relationship was observed.

Obesity might mediate the effect of meat consumption on depression [14, 15]. The mechanism how meat consumption increases the risk of depression might be speculated as follows. As a major component in meat, fat was considered to be associated with depression in animals. Abildgaard showed that rat is more susceptible to the development of depressive behavior following metabolic stress induced by high-fat diet [28]. Sumaya confirmed that high-fat diet may counteract the anti-depressive-like effect of the fluoxetine and reverse the anti-depressive-like effect of the 2-methyl-5-HT in mice [29]. Logically, a similar effect may exist in human. On the other hand, trans-unsaturated fatty acid, which was abundant in ruminant meat [30, 31], was found to be related to depression by Sanchez-Villegas [32]. However, some issues should also be noted. Firstly, no significant relationship between meat consumption and depression was found regarding the overall OR. Secondly, meat was complex in components and should be considered as a whole. However, there was no experimental study examining the effect of meat on depression directly. In addition, interestingly, some potential confounding factors might mediate the association between meat consumption and depression. Consuming less meat might be associated with some eating habits (vegetable and fruit consumption, legume consumption, et al.) [17] or lifestyle habits (physical activity) [33] that have a beneficial impact on the prevention of the risk of depression, which make it difficult to know in what proportion of our results should be attributed to other factors (vegetable and fruit consumption, legume consumption, physical activity, et al.). Therefore, more well-designed studies are needed to elaborate the concerned issues further.

Generally speaking, the muscle meat from beef, veal, pork, lamb, horse and deer is regarded as “red” meat. “White” meat mainly refers to poultry. “Processed meat” includes all types of meat products, such as sausages, cold cuts and other forms of meat, which have been mixed with other ingredients, such as salt, to extend their shelf life [8]. Therefore, it is speculated that the effect of meat may vary among various varieties. According to the epidemiological data, Wu confirmed that the effect of white meat, red meat and processed meat on hypertension differed greatly [34]. Becerra-Tomas showed that red or processed meat was associated with a higher risk of metabolic syndrome while white meat was not [35]. In terms of animal studies, Jakobsen found that the intake of red and white meat might lead to metabolic differences in rats [36]. Toden demonstrated that dietary red meat can cause a greater level of colonic DNA double-strand break than white meat in rats [37]. Therefore, it was speculated in the present study that the effect of white, red and processed meat on depression may differ from each other as well. An earlier meta-analysis found a relationship between processed meat and oral cancer, but this relationship did not exist when the meat was regarded as a whole [38]. Another meta-analysis showed that the processed or red meat was associated with cardiovascular disease while the total meat was not [39]. Hence, the present study attempted to specify the varieties as above. However, since no study has specified the varieties of meat with respect to depression yet, the various varieties could only be regarded as a whole. That means the results of this study are subject to the combined effect of different varieties of meat. Interestingly, the results for the overall RR and OR were totally different. Based on the overall RR, it was speculated that meat consumption might be associated with a moderately higher risk of depression indeed. However, depressive subjects may consume less meat due to the reduction in appetite, which might partly explain the reason why no significant relationship was established according to the overall OR. More well-designed prospective studies, which classify the different varieties of meat, are therefore needed.

The strengths of this meta-analysis can be listed as follow: Firstly, this is the first meta-analysis of observational study which aims at the relationship between meat consumption and depression. Secondly, the included studies were analyzed according to the adjusted results and large samples. Thirdly, the present study can serve as a reference and indication for further research. Nevertheless, this study also has several limitations. First, the results of this study might be distorted by the substantial level of heterogeneity. Second, since the relevant literature is limited, only a small number of studies were applicable for this meta-analysis. Third, the food frequency questionnaire, diagnostic criteria of depression and the selection of adjusted factors were not uniform. Fourth, few study specified the varieties of meat. Last but not the least, since some potential confounding factors might mediate the relationship between meat consumption and depression, some issues could not be addressed. As a consequence, the significance of this study might be weaken by the limitations above.

## Conclusions

The current evidences showed that meat consumption may be associated with a moderately higher risk of depression. However, due to the limited number of prospective studies and the potential confounding factors, it still warrants further studies with classification of meat varieties to confirm such findings.

## Additional file

Additional file 1:
**Table S1.** The methodological quality of cross-sectional studies in accordance with the Newcastle-Ottawa Scale (NOS). **Table S2.** The methodological quality of cohort studies in accordance with the Newcastle-Ottawa Scale (NOS). **Table S3.** The methodological quality of case-control studies in accordance with the Newcastle-Ottawa Scale (NOS). (DOCX 26 kb)

## Abbreviations

CI: Confidence intervals
NOS: Newcastle-Ottawa Scale
OR: Odds ratio
PRISMA: Preferred Reporting Items for Systematic review and Meta-analysis
RR: Relative risk
