# Supplementary material for: Is meat consumption associated with depression? A meta-analysis of observational studies
Source: BMC Psychiatry. 2017 Dec 28;17:409. doi: 10.1186/s12888-017-1540-7 (PMC5745880; doi:10.1186/s12888-017-1540-7)
Supplement: Additional file 1: — Table S1. The methodological quality of cross-sectional studies in accordance with the Newcastle-Ottawa Scale (NOS). Table S2. The methodological quality of cohort studies in accordance with the Newcastle-Ottawa Scale (NOS). Table S3. The methodological quality of case-control studies in accordance with the Newcastle-Ottawa Scale (NOS). (DOCX 26 kb) [file 12888_2017_1540_MOESM1_ESM.docx]

Additional file 1

Table S1 The methodological quality of cross-sectional studies in accordance with the Newcastle-Ottawa Scale (NOS)

| Study | Design | Data  collection | Response rate | Representativeness | Object and method | Power of testing | Statistical method | Total score |
| --- | --- | --- | --- | --- | --- | --- | --- | --- |
| Chen 2005 [16] | 1 | 1 | 1 | 1 | 1 | 0 | 1 | 6 |
| Miyake 2013 [21] | 1 | 1 | 1 | 0 | 1 | 0 | 1 | 5 |
| Zhou 2014 [22] | 1 | 1 | 1 | 1 | 1 | 0 | 1 | 6 |

Table S2 The methodological quality of cohort studies in accordance with the Newcastle-Ottawa Scale (NOS)

| Study | Selection | | | | Comparability | Outcome | | | Total score |
| --- | --- | --- | --- | --- | --- | --- | --- | --- | --- |
|  | Representativeness of the exposed cohort | Selection of the non-exposed cohort | Ascertainment of exposure | Demonstration that outcome of interest was not present at start of study | Comparability of cohorts on the basis of the design or analysis | Assessment of outcome | Was follow-up long enough for outcomes to occur? | Adequacy of follow up of cohorts? |  |
| Sanchez-Villegas 2009 [17] | 0 | 1 | 1 | 1 | 1 | 1 | 1 | 0 | 6 |
| Tsai 2011 [18] | 1 | 1 | 1 | 1 | 1 | 1 | 1 | 1 | 8 |
| Rienks 2013 [20] | 1 | 1 | 1 | 1 | 1 | 1 | 1 | 1 | 8 |

Table S3 The methodological quality of case-control studies in accordance with the Newcastle-Ottawa Scale (NOS)

| Study | Selection | | | | Comparability | Exposure | | | Total score |
| --- | --- | --- | --- | --- | --- | --- | --- | --- | --- |
|  | Is the case definition adequate | Representativeness of the cases | Selection of Controls | Definition of Controls | Comparability of cases and controls on the basis of the design or analysis | Ascertainment of exposure | Same method of ascertainment for cases and controls | Non-Response rate |  |
| Park 2012 [19] | 1 | 1 | 0 | 1 | 1 | 1 | 1 | 0 | 6 |
| Kim  2015 [23] | 1 | 1 | 1 | 1 | 1 | 1 | 1 | 0 | 7 |
